# Population genomics and phylogeography of *Colletes gigas*, a wild bee specialized on winter flowering plants

**DOI:** 10.1002/ece3.8863

**Published:** 2022-04-24

**Authors:** Tianjuan Su, Bo He, Fang Zhao, Kai Jiang, Gonghua Lin, Zuhao Huang

**Affiliations:** ^1^ 105809 School of Life Sciences Jinggangshan University Ji'an China; ^2^ 12514 College of Life Sciences Anhui Normal University Wuhu China

**Keywords:** colletid bees, demographic history, genetic variation, population genomics, population structure, specialist pollinator

## Abstract

Diet specialization may affect the population genetic structure of pollinators by reducing gene flow and driving genetic differentiation, especially in pollen‐specialist bees. *Colletes gigas* is a pollen‐specialist pollinator of *Camellia oleifera*, one of the most important staple oil crops in China. *Ca*. *oleifera* blooms in cold climates and contains special compounds that make it an unusable pollen source to other pollinators. Thus, *C*. *gigas* undoubtedly plays a key role as the main pollinator of *Ca*. *oleifera*, with biological and economic significance. Here, we use a population genomic approach to analyze the roles of geography and climate on the genetic structure, genetic diversity, and demographic history of *C*. *gigas*. A total of 1,035,407 SNPs were identified from a 582.77 Gb dataset. Clustering and phylogenetic analyses revealed a marked genetic structure, with individuals grouped into nine local clusters. A significant isolation by distance was detected by both the Mantel test (*R* = .866, *p* = .008) and linear regression (*R* ^2^ = .616, *p* < .001). Precipitation and sunshine duration were positively and significantly (*R* ≥ .765, *p* ≤ .016) correlated with observed heterozygosity (*H*_o_) and expected heterozygosity (*H*_e_). These results showed that *C*. *gigas* populations had a distinct phylogeographic pattern determined by geographical distance and environmental factors (precipitation and sunshine duration). In addition, an analysis of paleogeographic dynamics indicated that *C*. *gigas* populations exhibited patterns of glacial expansion and interglacial contraction, likely resulting from post‐glacial habitat contraction and fragmentation. Our results indicated that the peculiar phylogeographic patterns in *C*. *gigas* populations may be related to their specialization under long‐term adaptation to host plants. This work improves our understanding of the population genetics in pollen‐specialist bees. The distinct genetic clusters identified in this study should be taken into consideration for the protection and utilization of this specialized crop pollinator.

## Graphical abstract

In this study, we use a population genomic approach to analyze genetic structure, genetic diversity, and demographic history of *Colletes gigas*, a pollen‐specialist pollinator of *Camellia oleifera*. A marked genetic structure and a significant isolation by distance effect were detected among populations. Precipitation and sunshine duration were positively and significantly correlated with population genetic heterozygosity. The historical population dynamics exhibited patterns of glacial expansion and interglacial contraction, likely resulting from post‐glacial habitat contraction and fragmentation. Our results indicated that the peculiar phylogeographic patterns in *C. gigas* populations may be related to their specialization under long‐term adaptation to host plants.

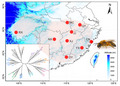

## 1 INTRODUCTION

Bees are widely recognized as key pollinators of both wild and cultivated plants; however, their populations are in decline, causing concern globally (Potts et al., 2016). The main determinants driving the bee population decline are thought to be habitat loss and fragmentation as well as climate change (Althausa et al., 2021; Hadley & Betts, 2012; Millard et al., 2021; Potts et al., 2010; Soroye et al., 2020). Habitat loss can lead to a reduction in effective population size (*N*_e_) and a loss of genetic diversity (Kennedy et al., 2013; Zayed et al., 2005). Habitat fragmentation may reduce genetic connectivity between populations, which results in inbreeding and genetic drift, thereby increasing genetic differentiation (Fischer & Lindenmayer, 2007; Jha, 2015; Jha & Kremen, 2013). In addition, climate change is expected to alter the availability of nesting and floral resources, leading to population reductions (Dellicour et al., 2015; Faleiro et al., 2018; Kerr et al., 2015; Pyke et al., 2016; Willmer, 2014).

Understanding the genetic structure of bee populations is the key to predicting their susceptibility to environmental change and is essential for conservation management (Grozinger & Zayed, 2020; López‐Uribe et al., 2017). Therefore, to maintain an effective and healthy pollinator service, it is important to understand how pollinator populations and communities respond to variable environments. A growing number of studies have shown that natural populations of wild and managed bees, especially pollen‐specialist species, are in rapid decline (range reduction and/or population decrease) around the world, raising concerns about the future of the ecosystem services of bees and their contribution to crop pollination (Biesmeijer et al., 2006; Burkle et al., 2013; Cameron et al., 2011; Garibaldi et al., 2013; Potts et al., 2010, 2016; Steffan‐Dewenter et al., 2005). Surprisingly, the effects of environmental change, including some anthropogenic activities, on bees are not always negative. Some species adapt to and even thrive in human‐dominated habitats. For example, habitat restoration promotes the rapid colonization of new habitats in cities and suburbs (Matteson et al., 2008; Theodorou et al., 2018), increasing the genetic diversity of bees (Ballare & Jha, 2020; Theodorou et al., 2020; Vickruck & Richards, 2017). In addition, the natural abundance and distribution of host plants (Dellicour et al., 2014, 2015) and the human‐mediated domestication of crops (López‐Uribe et al., 2016) can promote the rapid expansion of obligate bees with respect to population size and geographic distribution.

As the largest *Colletes* bee species in the world, *Colletes gigas* is a pollen specialist and is endemic to China (Niu et al., 2013). It is genetically, morphologically, and ecologically distinct from other colletid species with different geographic distributions and floral choices (Niu et al., 2014). *C*. *gigas* is the main pollinator of *Camellia oleifera*, a major woody oil plant in China (Li et al., 2021). Although some other insects, such as *Andrena* spp., *Vespa bicolor*, and *Phytomia zonata*, visit *Ca*. *oleifera* (Li et al., 2021; Wei et al., 2019), *C*. *gigas* is the most important pollinator able to detoxify *Ca*. *oleifera*. Notably, this solitary univoltine bee nests underground, with its reproductive activity consistent with the flowering period of *Ca*. *oleifera*. However, *Ca*. *oleifera* presents a low oil yield because of self‐incompatibility. The oil yield can be increased by an increase in pollinating insects (Deng et al., 2010; Li et al., 2021) and optimal cross‐pollination combinations (Hu et al., 2020). *Ca*. *oleifera* blooms from autumn to winter (from October to January), during which bee pollinators are quite limited because temperatures are low. In addition, compounds in the pollen and/or nectar are toxic to most other bees, including managed honeybees (Xie et al., 2013). Accordingly, the product yield of camellia oil is currently in short supply because of low pollination services from these bee pollinators. However, both adults and larvae of *C*. *gigas* can detoxify the toxic components in *Ca*. *oleifera* (Zhou et al., 2020). Accordingly, *C*. *gigas* became an important pollinator for *Ca*. *oleifera* and has attracted substantial attention (Deng et al., 2010; Huang et al., 2016, 2017; Li et al., 2021; Zhou et al., 2020). *Ca*. *oleifera* is one of the most important staple oil crops in China, with a cultivated area exceeding 4.67 million hectares (Wen et al., 2018). *Ca*. *oleifera* oil was listed by the Food and Agriculture Organization of the United Nations (FAO) as a premium health‐grade edible oil (Feng et al., 2020). However, there is a contradiction between the accelerating industrialization of *Ca*. *oleifera* and the habitat degradation of pollinators. Given the biological and economic importance of *C*. *gigas*, a clear understanding of its population dynamics and the implementation of corresponding measures to protect this key pollinator are urgently needed. However, such conservation measures would require the prior determination of the spatial distribution of genetic variation. The draft genome of *C*. *gigas* has been sequenced, providing a powerful toolset for studies of population genetic structure (Zhou et al., 2020). These data will also be helpful for assessments of the impact of habitat loss on functional connectivity and genetic diversity in bees, analyses of adaptation to local environmental conditions, and the future management of bee populations.

In this study, 55 samples from nine regions were collected from the main distribution of *C*. *gigas* populations. The whole genomes were resequenced to clarify the population structure, genetic diversity, and demographic history, as well as the impacts of environmental factors on genetic variation. In particular, we investigated (a) regional‐scale population structure and differentiation, (b) genetic diversity in local populations, and (c) the population history and relationships with environmental factors, including precipitation, sunshine duration, and temperature to obtain insight into climate‐driven demography and the demographic stability of this important crop pollinator. We also explored whether these climatic variables were helpful in explaining the genetic structure observed in *C*. *gigas* populations. This study provides new insights into adaptive genetic variation in this specialist bee and the roles of environmental variables and host plants in its evolution. Additionally, the results provide an important reference for predicting bee survival and for crop pollinator management.

## 2 MATERIALS AND METHODS

### 2.1 Specimen collection

**FIGURE 1:**
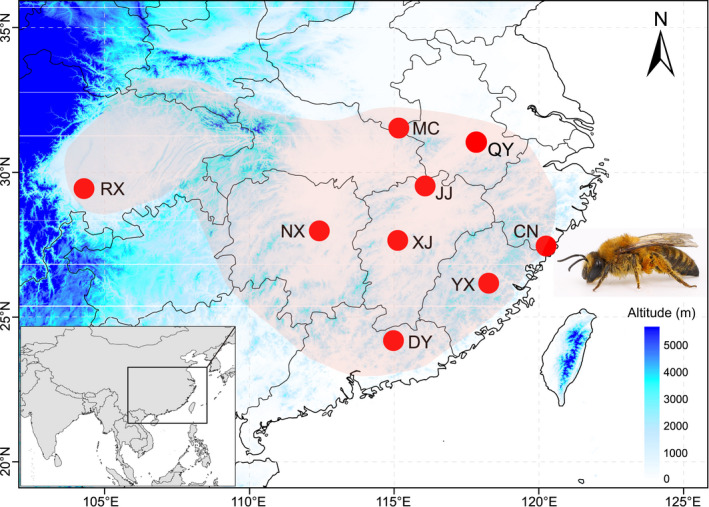
Map of the sampling sites and the geographic distribution of *Colletes gigas*. Sample localities (circles) and current distribution (light red shading). Populations are defined as follows: DY, Dongyuan; YX, Youxi; CN, Cangnan; XJ, Xiajiang; JJ, Jiujiang; QY, Qinyang; MC, Macheng; NX, Ningxiang; RX, Rongxian

In the vast majority of cases, the female and male bees are diploid and haploid, respectively. Their genetic characteristics are quite different, especially at the genomic level (Grozinger & Zayed, 2020; Zayed, 2009). Given that the current mainstream methods for population genome analysis are performed on diploid data (see Ballare & Jha, 2020; Ji et al., 2020), female specimens were used to ensure data consistency. Female adults of *C*. *gigas* (*N* = 55) were collected between October and December in 2019 and October in 2020, using a systematic sampling strategy to ensure the uniformity of spatial distribution (Table 1, Figure 1). All specimens were identified based on both morphological characteristics (refer to Niu et al., 2013) and COI gene data from the National Center for Biotechnology Information (NCBI) (Text S1, Table S1, Figure S1). In addition, the voucher specimen of MC (China: Hubei, Macheng) was preserved at the Entomological Specimen Room of Jinggangshan University (accession number: 20191110MC01). Five to nine females were selected from each population. All samples were collected using a sweep net from the oil tea camellia flowers. Each individual was stored in absolute ethanol and then frozen at –20°C until genomic DNA extraction.

**TABLE 1:** *Colletes gigas*population genetic diversity and sample data

Code	Location	Date	Longitude	Latitude	*N*	*H*_o_	*H*_e_
DY	Dongyuan (Guangdong)	30‐Nov‐19	114.9792	24.1905	6	0.255	0.253
YX	Youxi (Fujian)	1‐Dec‐19	118.2649	26.1719	6	0.251	0.234
CN	Cangnan (Zhejiang)	2‐Nov‐19	120.2556	27.4591	6	0.239	0.228
XJ	Xiajiang (Jiangxi)	3‐Dec‐19	115.1285	27.6546	5	0.231	0.198
JJ	Jiujiang (Jiangxi)	21‐Nov‐19	116.0748	29.5333	6	0.220	0.201
QY	Qinyang (Anhui)	29‐Oct‐20	117.8796	30.5977	6	0.231	0.203
MC	Macheng (Hubei)	10‐Nov‐19	115.1678	31.5303	9	0.236	0.208
NX	Ningxiang (Hunan)	19‐Nov‐19	112.4206	27.9832	6	0.190	0.184
RX	Rongxian (Sichuan)	14‐Nov‐19	104.2913	29.4377	5	0.105	0.079

Note

*N* is the number of individuals analyzed from each collection.

Abbreviations: *H*_e_, expected heterozygosity; *H*_o_, observed heterozygosity.

### 2.2 Whole‐genome resequencing and SNP calling

Total genomic DNA was extracted from the thorax of each individual using the QIAGEN DNeasy Blood and Tissue kit (Germany), following the manufacturer's protocols. DNA libraries with ~350 bp insertions were constructed. Then, a genomic library with an insert size of 150 base pairs was constructed. Genome resequencing for each sample was performed using the Illumina HiSeq 2000 sequencing platform (Shi et al., 2020). Quality control for raw sequence data was performed using fastp 0.20.0 (Chen et al., 2018) with the parameters “‐q 15 ‐n 10 ‐u 40.” Both paired reads were filtered out if either one contained over 40% of low‐quality bases or more than 10% Ns. Adapter contamination was also trimmed. Samples were sequenced to a target depth of ~35–53× (ca. 9.5 to 14.3 Gb of clean data per sample). The clean sequence data for all 55 individuals have been submitted to the National Center for Biotechnology Information (NCBI) under project PRJNA768656.

To detect population‐level SNPs, clean reads were mapped to the *C*. *gigas* reference genome (GCA_013123115.1_ASM1312311v1_genomic.fna, genome size: 273.06 Mb, N50 = 8.11 Mb) (Zhou et al., 2020) using Burrows‐Wheeler Alignment (BWA) 0.7.12 (Li & Durbin, 2009). Alignments were transformed to BAM files using SAMtools 1.3 (Li et al., 2009). The HaplotypeCaller method implemented in Genome Analysis Toolkit (GATK) 4.1.3.0 (McKenna et al., 2010) was used for SNP calling across the 55 individuals. SNPs with an allele frequency of <20% and with a depth distribution of all sites of <2.5% or >97.5% were filtered using a custom script to obtain high‐quality SNPs. Moreover, low‐quality SNPs were filtered out when the base quality and mapping quality score was <20. Then, a Python script (https://github.com/JingfangSI/SnpCountCU/) was used to count the number of SNPs that are unique within populations and common among populations from a VCF format file.

### 2.3 Genetic diversity and differentiation

The population structure was calculated using ADMIXTURE 2.3.4 (Alexander et al., 2009), with ancestral clusters (*K*) ranging from 2 to 9. The best *K* value was identified based on the cross‐validation procedure. A covariance matrix calculated from genotype likelihoods of SNPs with PLINK2 (Purcell et al., 2007) was used to perform the principal component analysis (PCA) with “prcomp” function in R 4.1.1. For the overall consensus phylogenetic tree, SNPhylo (Lee et al., 2014) was used. Before tree construction, SNPs were filtered with missing rate >0.1, minor allele frequency (MAF) <0.05, and a linkage disequilibrium (LD) threshold of 0.2. In total, 12,566 high‐quality SNPs were finally used to construct the maximum likelihood tree using SNPhylo with default parameters and 1000 bootstrap replicates.

For the population genetic analysis, a Bayesian *F*_ST_ outlier test in BayeScan 2.1 (Foll & Gaggiotti, 2008) was performed, with the q‐value threshold of 0.05, after running for 5000 outputted iterations with 50,000 burn‐in and retaining every 10th iteration. Finally, we removed any potential loci (1.6%) from the neutral dataset. Subsequently, VCFtools (Danecek et al., 2011) was used to analyze the neutral loci with a window size of 10,000 SNPs. Pairwise nucleotide variation was estimated as a measure of genetic differentiation (*F*_ST_). Theoretically, *F*_ST_ cannot be less than 0, but sometimes calculations through software still give a small number of negative values. We set these negative *F*_ST_ estimates to 0, which means that there is no genetic subdivision between the populations considered (Massardo et al., 2020). The genetic diversity indices observed that heterozygosity (*H*_o_) and expected heterozygosity (*H*_e_) were calculated for each population using PLINK2 (Purcell et al., 2007), with a window size of 10,000 SNPs. The historical effective population size (*N*_e_) was calculated using SMC++ (Terhorst et al., 2017) with the mutation rate set to 3.6 × 10^−9^ (Liu et al., 2017) and the generation time (g) to 1 year.

### 2.4 Effects of climate on genetic variation

The geographic distance (in km) was calculated using the ArcGIS platform. The Mantel test of the geographic distance and genetic distance (*F*_ST_) was performed using the ade4 package in R (Dray & Dufour, 2007). Furthermore, we performed a linear regression analysis between genetic distance (standardized by *F*_ST_/(1−*F*_ST_)) and geographical distance (log_10_ transformed). Precipitation, sunshine duration (i.e., the time of effective solar radiation during the day without cloud cover), and average temperature were considered as the most effective predictors of bee ranges (Jackson et al., 2018; Koch et al., 2019). We obtained these surface meteorological data from 2010 to 2016. The Pearson's correlation coefficients were evaluated for the relationships between the three climate variables and genetic diversity (*H*_o_ and *H*_e_).

## 3 RESULTS

**FIGURE 2:**
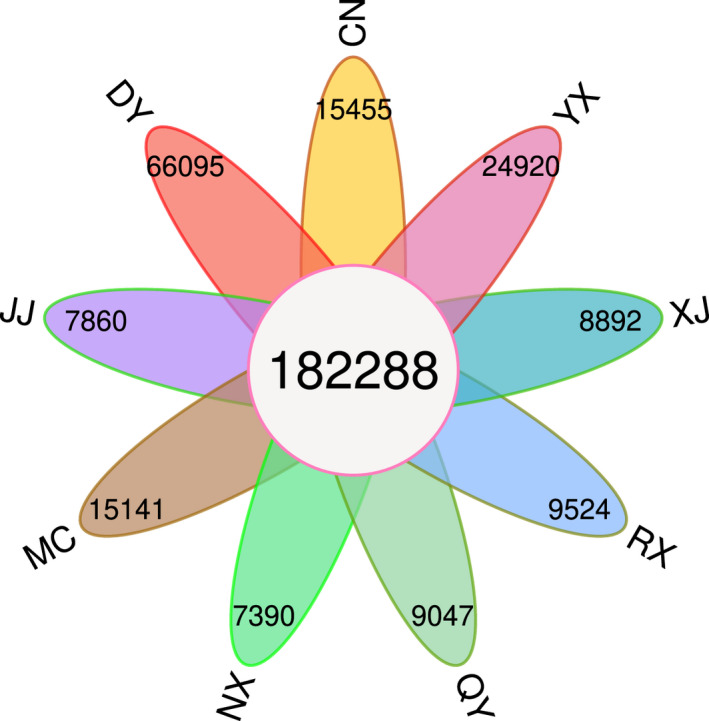
Flower diagram showing the numbers of specific SNPs per population and common SNPs across the nine populations of *Colletes gigas*. Populations are defined as follows: DY, Dongyuan; YX, Youxi; CN, Cangnan; XJ, Xiajiang; JJ, Jiujiang; QY, Qinyang; MC, Macheng; NX, Ningxiang; RX, Rongxian

We obtained genomic data for 55 individuals of the wild bee *C*. *gigas*, specialized in feeding and pollinating *Ca*. *oleifera*, from nine populations in China (Table 1). Whole‐genome resequencing yielded 582.77 Gb of sequence data. The average coverage depth of clean data was approximately 38.8× (Table S2). With the GATK SNP calling strategy, 1,035,407 SNPs were identified for further analysis. The number of common/shared SNPs was 182,288 (17.61%), and the number of specific SNPs per population ranged from 7390 (0.76%) to 66,095 (6.38%) (Figure 2).

### 3.1 Population structure

**FIGURE 3:**
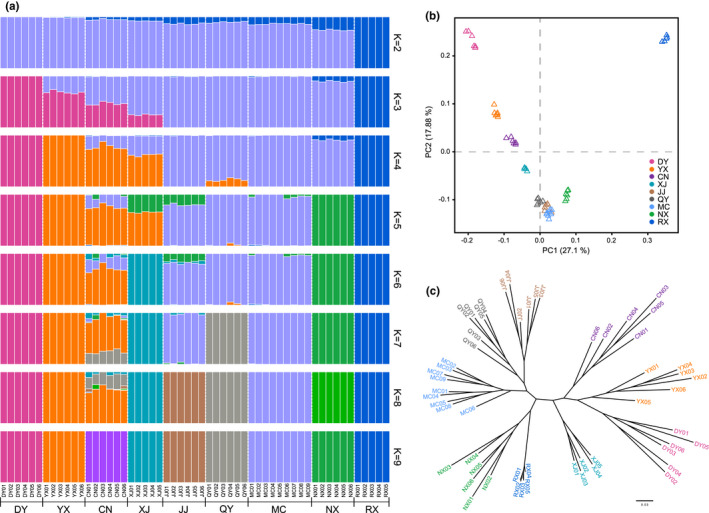
Population structure results for *Colletes gigas*. (a) ADMIXTURE results based on whole‐genome SNPs with *K* = 2 to 9. (b) PCA of all individuals in the space of the first two principal components from nine populations. (c) Maximum likelihood phylogenetic tree generated using SNPhylo. In both plots, populations are defined as follows: DY, Dongyuan; YX, Youxi; CN, Cangnan; XJ, Xiajiang; JJ, Jiujiang; QY, Qinyang; MC, Macheng; NX, Ningxiang; RX, Rongxian

A cluster analysis was performed using ADMIXTURE to examine the genetic relationships among populations. With a *K* value of 2, *C*. *gigas* in the RX region was divided into one class, while the other regions formed a large cluster with clear boundaries in adjacent regions (Figure 3a). As *K* increased from 3 to 4, DY, YX, and RX presented distinct ancestries from other populations. JJ, QY, MC, and NX showed different levels of admixture. For a *K* value of 5 to 7, the differentiation of XJ, QY, and NX was further emphasized. When the *K* value was 8, we observed the stratification of eight populations; however, CN was mixed with YX and slightly with other populations (such as QY, XJ, JJ, and NX). When the *K* value was 9, CN finally showed independent status from other populations. We also explored the relationship of the species using PCA performing on the genetic covariance matrix among all individuals. The PCA results showed a considerable degree of interpopulation differentiation, with most species formed distinct point clusters in the space of the first two principal components (proportion of the total variance explained: *PC1* 27.1% and *PC2* 17.88%, Figure 3b). Significantly, the RX population was more separated, further demonstrating its isolation. We then constructed a maximum‐likelihood tree using a subset of 12,566 high‐quality SNPs to identify the phylogenetic relationships among nine populations (Figure 3c). Although there were still some low support values, the results showed that each population was monophyletic. In conclusion, these results consistently indicated that the differentiation of *C*. *gigas* was significant. It was worth mentioning that the CV errors of ADMIXTURE increased with the *K* values without an inflection point (Table S3); we believe that *K* = 2 presents the ideal stratification. PCA results also showed that the RX population was more separated, reflecting a high isolation level from the other populations.

### 3.2 Population differentiation

**FIGURE 4:**
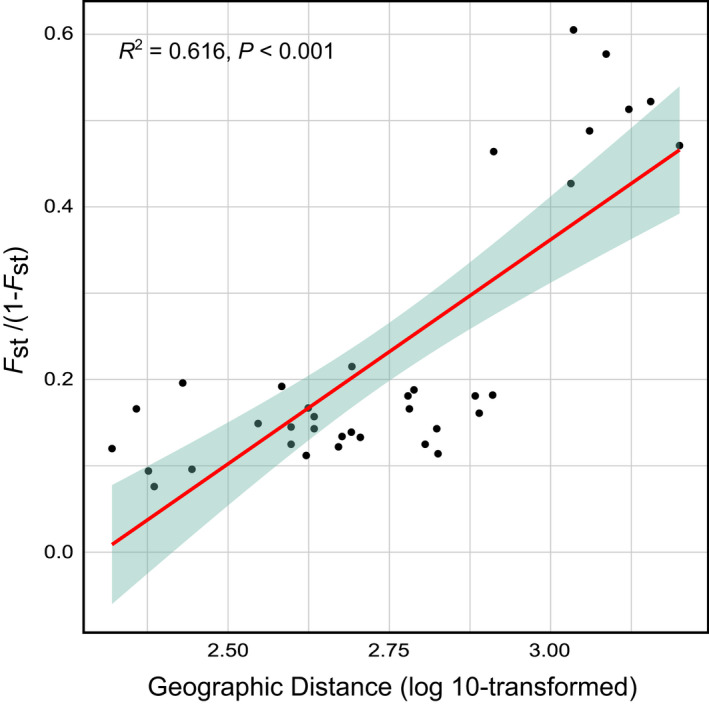
The relationship between genetic distance (*F*_ST_/(1−*F*_ST_)) and geographical distance (log_10_ transformed)

The pairwise *F*_ST_ values among populations were calculated to quantify genetic differentiation (Table 2). Pairwise *F*_ST_ ranged from 0.071 (between CN and YX) to 0.377 (between XJ and RX), with an average of 0.174. *F*_ST_ between RX and the other populations ranged from 0.299 to 0.377 (mean *F*_ST_ = 0.336), much higher than those among the other populations, indicating elevated genetic differentiation of RX from the other populations. There was very limited genetic differentiation among other populations (*F*_ST_ < 0.2), suggesting that gene flow was common among these populations. To further investigate the influence of geographic distance on divergence, we evaluated the correlation between the geographic distance matrix and pairwise *F*_ST_ matrix. The Mantel test showed a significant positive correlation between geographic distances and genetic distances (*R* = .866, *p* = .008). Similarly, linear regression showed a significant correlation between *F*_ST_/(1−*F*_ST_) and geographical distance (Log_10_ transformed) (*R* ^2^ = .616, *p* < .001, Figure 4). These results indicated that isolation by distance was a significant factor driving population differentiation of *C*. *gigas*.

**TABLE 2:** Pairwise *F*_ST_ values below the diagonal and Euclidean geographic separation (km) above the diagonal

	DY	YX	CN	XJ	JJ	QY	MC	NX	RX
DY	–	396	639	383	601	764	813	492	1219
YX	0.111	–	243	352	430	491	666	614	1429
CN	0.111	0.071	–	507	469	418	669	775	1584
XJ	0.161	0.130	0.117	–	228	421	430	269	1085
JJ	0.153	0.125	0.109	0.142	–	209	238	396	1149
QY	0.153	0.122	0.101	0.143	0.107	–	278	604	1322
MC	0.154	0.125	0.102	0.136	0.086	0.088	–	475	1075
NX	0.177	0.158	0.139	0.164	0.127	0.142	0.118	–	816
RX	0.366	0.343	0.320	0.377	0.328	0.339	0.299	0.317	–

### 3.3 Genetic diversity and climate effects

The mean observed heterozygosity (*H*_o_) and mean expected heterozygosity (*H*_e_) were 0.218 (0.105–0.255) and 0.199 (0.079–0.253), respectively (Table 1). Overall, we found that *H*_o_ was greater than *H_e_* at the population level. There were significant differences (*p* < .01) in *H*_o_ and *H*_e_ among the nine populations. DY had the highest heterozygosity, while RX had the lowest heterozygosity. The high heterozygosity among populations might be explained by the combined effects of mutation, selection, and genetic drift. We found that the genetic diversity of *C*. *gigas* decreased from southeast to northwest and/or north with respect to the geographical distribution. The correlations between genetic diversity (*H*_o_ and *H*_e_) and environmental factors (precipitation, sunshine duration, and temperature) were analyzed. Precipitation was positively correlated with *H*_o_ (*R* = .830, *p* = .006) and *H*_e_ (*R* = .765, *p* = .016). Sunshine duration was also positively correlated with *H*_o_ (*R* = .822, *p* = .006) and *H*_e_ (*R* = .831, *p* = .006). However, there was no correlation between genetic diversity and temperature (*H*_o_, *p* = .370; *H*_e_, *p* = .220). These results indicated that genetic diversity was higher in areas with more precipitation and sunshine duration, where gene flow in *C*. *gigas* was also more frequent. Accordingly, the clinal changes in genetic diversity were consistent with geographical patterns of precipitation and sunshine duration.

### 3.4 Demographic history

**FIGURE 5:**
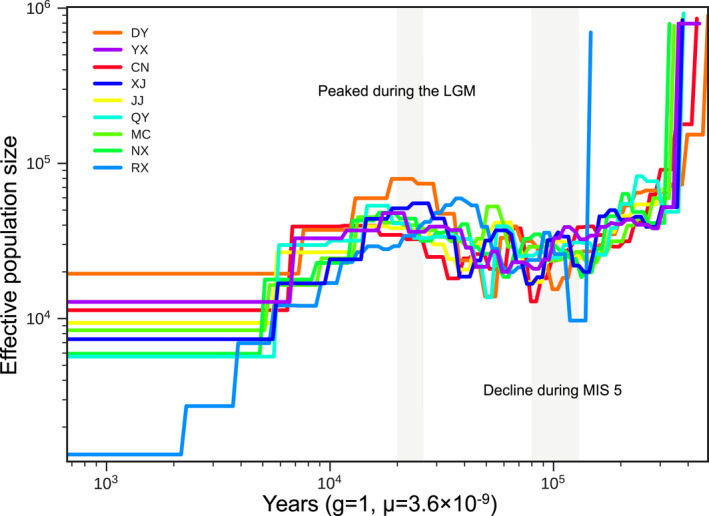
Dynamics of effective population sizes (*N*_e_) inferred by SMC++, with a generation time (g) of 1 year and a mutation rate (*µ*) of 3.6 × 10^−9^ per site per generation. Populations are defined as follows: DY, Dongyuan; YX, Youxi; CN, Cangnan; XJ, Xiajiang; JJ, Jiujiang; QY, Qinyang; MC, Macheng; NX, Ningxiang; RX, Rongxian

To explore the demographic history of *C*. *gigas*, the historical effective population sizes (*N*_e_) were estimated using SMC++. All populations underwent multiple changes in population size during their evolutionary history (Figure 5). The species experienced an obvious population decline approximately ~0.4 Ma, coinciding with the glacial–interglacial cycles (Jouzel et al., 2007; Kawamura et al., 2007). The most dramatic decline in *N*_e_ occurred during Marine Isotope Stage 5 (MIS5, 80–130 ka ago) (Jouzel et al., 2007; Lisiecki & Raymo, 2005). Then, in the Last Glacial Period (LGP), *N*_e_ increased continuously and peaked during the Last Glacial Maximum (LGM, ~20 ka ago) (Clark et al., 2009). Conversely, after LGM *N*_e_ began to decline in the current interglacial period. Notably, *N*_e_ for RX population had a higher standard deviation (0.88) than those of the other populations (0.32–0.51), indicating that RX had a higher demographic fluctuation. Moreover, RX showed the greatest decline among the nine populations since the LGM, which may reflect the high sensitivity of this population to climatic events and a lack of gene flow with surrounding populations, thus forming a strongly isolated population.

## 4 DISCUSSION

Overall, the SNP analyses of nine population pools of *C*. *gigas* revealed a marked genetic structure. First, a cluster analysis based on ADMIXTURE showed that there was very limited shared genetic variation between RX and any other population. Except for CN and YX, all populations were relatively independent and only showed minor shared variation. Second, the phylogeny based on individuals showed that all nine populations formed independent monophyletic clades, indicating significant genetic differentiation among *C*. *gigas* populations. Third, pairwise *F*_ST_ among populations was positively correlated with geographical distance, suggesting that geographical distance had a significant effect on the genetic differentiation among populations of *C*. *gigas*. The three analyses consistently proved that there is substantial genetic structure and differentiation among the populations of *C*. *gigas*.

Body size in bees is an important predictor of genetic differentiation, with larger species exhibiting wider foraging ranges and less differentiation (López‐Uribe et al., 2019). Interestingly, however, it may not be beneficial for *Colletes* bees, which usually have weak migration ability. For example, both *Colletes floralis* (females: 9–12 mm body length) (Davis et al., 2010) and *Colletes hederae* (females: 10–13 mm body length) (Dellicour et al., 2014) showed obvious genetic structure over a similar geographical scale to that in this study. As the largest species of *Colletes*, *C*. *gigas* (females: 17–18 mm body length) (Niu et al., 2013) might have relatively strong migration ability, in theory. However, in this study, we detected significant genetic differentiation among *C*. *gigas* populations. The genetic structure may be related to their specialization under long‐term adaptation to host plants. Previous studies have revealed that the availability of flowers is a key factor affecting population differences and genetic variation (Dellicour et al., 2015; Kahnt et al., 2014, 2018). Foraging in *C*. *gigas* occurs in the autumn and winter (from October to January), consistent with the flowering season for its exclusive host plant. It is obvious that the low temperature in the two seasons is not conducive to foraging activity and long‐distance flight. Additionally, *C*. *gigas* depends on the pollen and nectar of *Ca*. *oleifera* for breeding (Zhao et al., 2010), given that the distribution of *Ca*. *oleifera* in nature is discontinuous (Huang et al., 2018), *C*. *gigas* can only rely on *Ca*. *oleifera* in local patches. Moreover, adults need to mate, build nests, and collect pollen and nectar over a short period of time, and these factors further force the species to rely heavily on nearby host plants. As a result, due to the specialized living habits, gene flow among *C*. *gigas* populations was largely limited, resulting in substantial genetic structure and differentiation among populations.

Genetic variation in oligolectic bees (especially specialist bees) is likely to be strongly affected by the geographical and temporal distributions of host plants (Dellicour et al., 2014, 2015; López‐Uribe et al., 2016; Zayed et al., 2005). In this study, a clinal change in genetic diversity was found across the distribution of *C*. *gigas*, with higher genetic diversity in populations in the southeast than in the northwest and/or north. Most interestingly, we found that there was a significant positive correlation between genetic diversity and precipitation and sunshine duration. Previous studies also found that precipitation has indirect (e.g., availability of floral resources) or even direct (e.g., desiccation pressure) influences on bee fitness (Maia‐Silva et al., 2015) and might be a highly effective predictor of bee ranges (Jackson et al., 2018; Koch et al., 2019). In China, *Ca*. *oleifera* is mainly distributed in the warm and humid areas of the subtropical zone (Liu et al., 2018). For the arid environment in the autumn, relatively more but discontinuous precipitation could prompt typical host plant flowering. Therefore, precipitation could indirectly affect the availability of floral resources for bees. Precipitation could also improve the soil humidity, which helps bees to dig underground nests (da Costa et al., 2019). Therefore, precipitation may help to meet the special nesting requirements for *C*. *gigas*. In winter, sunlight had the most direct effect on both the flowering of *Ca*. *oleifera* and the foraging behavior of *C*. *gigas*. Although low temperature could force bees to forage under adverse weather conditions, reduce the foraging range and gene flow, and lead to genetic differentiation between populations (Jaffé et al., 2019; Kahnt et al., 2018; Linder et al., 2010), there was no correlation between genetic diversity and temperature in this study. It is possible that the effect of temperature on *C*. *gigas* populations was offset by temporal niches. *Ca*. *oleifera* is induced by low temperature and blooms earlier in regions with much lower temperatures (Jiang et al., 2017). In fact, our field observations also revealed that the emergence time of *C*. *gigas* was synchronized with the flowering phenology of *Ca*. *oleifera* at different sampling sites. Overall, precipitation and sunshine duration were the major factors that directly and/or indirectly influenced genetic diversity in *C*. *gigas* populations.

A clear signal of past population growth was found based on genome‐wide site frequency spectra. We found that the *N*_e_ decreased with increasing global temperature (e.g., during the last major interglacial period) but increased with decreasing global temperature (e.g., during the LGM). This pattern suggested that global temperature had a strong effect on the effective population sizes of *C*. *gigas*. A markedly different pattern has been observed for the Quaternary paleogeographic dynamics for many other bee species. These bee populations experienced sharp declines during the last ice age, followed by rapid expansion and species diversification from glacial refugia after the end of the last ice age (Dellicour et al., 2015; Dew et al., 2016; Groom et al., 2014; Shell & Rehan, 2016). However, our results suggested that low temperatures were more favorable for *C*. *gigas* survival, which might be related to the fact that it is a winter‐active species with a high tolerance to cold climates. Thus, *C*. *gigas* exhibited interglacial contractions and glacial expansions, similar to the dynamics of other cold‐adapted bees typical of ice ages, e.g., *Apis mellifera sinisxinyuan* n. ssp. (Chen et al., 2016), *Anthophora plumipes* (Černá et al., 2017), and some bumblebees (Dellicour et al., 2017). In the interglacial periods, *C*. *gigas* populations were small, suggesting that they were in refugia due to inappropriate habitat conditions. These findings implied that host flower production was very low after the ice age.

Additionally, a clinal change in *N*_e_ was found across the distribution of *C*. *gigas*, which was also consistent with the pattern of genetic diversity (i.e., *N*_e_ was higher in the southeast than in the northwest and/or north). This finding suggested that the southeastern populations had more time to accumulate higher genetic variation under suitable environmental conditions. However, it is interesting to note that *N*_e_ for the RX population had a higher standard deviation than those of the other populations, suggesting that RX had a higher demographic fluctuation. Although there was some delay during the glacial period, the precise reasons are unknown. This population suffered its worst decline since LGM and was smaller than all other populations. This might be explained by a severe bottleneck when the population declined and became an isolated group. Small, isolated populations often present an increased risk of extinction due to various intrinsic factors, such as inbreeding and genetic drift (Allendorf & Luikart, 2006; Laikre et al., 2010). Therefore, such small, highly structured populations like RX need to be protected. To avoid the loss of genetic diversity in *C*. *gigas*, we should also take measures to prevent habitat fragmentation or connect isolated patches in the future cultivation and management of *Ca*. *oleifera* (Huang et al., 2018). In addition, the genetic structure of bee populations is also related to the temporal distribution and diffusion of host plants (López‐Uribe et al., 2016; Vickruck & Richards, 2017). Different from most other crop pollination bees, *C*. *gigas* is very specific to *Ca*. *oleifera*. Theoretically, the stability of *C*. *gigas* population is likely to be affected by the stability of *Ca*. *oleifera* population. Previous studies had shown that the demographic history of *Ca*. *oleifera* (wild *Ca*. *oleifera*) is relatively stable in the subtropical region, but present much larger fluctuation in the northwest (Cui et al., 2016; Liu et al., 2018). This is consistent with the genetic diversity of *C*. *gigas* population observed in our study. It should be pointed out that in recent years, the cultivation of *Ca*. *oleifera* has received unprecedented attention and promotion efforts from the government. Although such policy is helpful to increase the amount of floral resources and the connectivity of landscape, intensively managed forestry would cause the habitat loss, which was not conducive to maintaining the population stability of *C*. *gigas* (Huang et al., 2017), which in turn affected the per‐unit yield of *Ca*. *oleifera*. The enhancement of intensive cultivation will cause habitat loss for crop pollinators, and ultimately affect the stability of pollination services (Deguines et al., 2014; Millard et al., 2021; Montoya et al., 2021; Potts et al., 2016). We believe that more attention should be paid to the contradiction between the intensification of *Ca*. *oleifera* cultivation and the degradation of the habitat of crop pollinators, so as to organically combine economic development with ecosystem health.

## CONFLICT OF INTEREST

The authors have no conflict of interest to declare.

## AUTHOR CONTRIBUTIONS

**Tianjuan Su:** Funding acquisition (equal); Methodology (equal); Software (equal); Writing – original draft (equal). **Bo He:** Methodology (equal); Writing – original draft (equal). **Fang Zhao:** Funding acquisition (equal). **Kai Jiang:** Resources (equal). **Gonghua Lin:** Methodology (equal); Software (equal); Writing – review & editing (equal). **Zuhao Huang:** Conceptualization (equal); Funding acquisition (equal); Resources (equal).

## Supporting information

Supplementary Material

## Data Availability

The data that support the findings of this study are openly available in [GenBank] at https://www.ncbi.nlm.nih.gov/bioproject/, accession PRJNA768656. Relevant data in this study will be available via Dryad: https://doi.org/10.5061/dryad.kkwh70s6v.
