# Supplementary material for: Population genomics and phylogeography of Colletes gigas, a wild bee specialized on winter flowering plants
Source: Ecol Evol. 2022 Apr 24;12(4):e8863. doi: 10.1002/ece3.8863 (PMC9035574; doi:10.1002/ece3.8863)
Supplement: Supplementary file 1 — Supplementary Material [file ECE3-12-e8863-s001.docx]

**Supplementary Information for:**

**Population genomics and phylogeography of Colletes gigas, a wild bee specialized on winter flowering plants**

**This PDF file includes:**

Supplementary Information Text

FIGURE S1

Supplementary Table 1

Supplementary Table 2

Supplementary Table 3

**Supplementary Information Text**

**DNA barcoding analyses of the genus** ***Colletes gigas***

The 55 COI gene sequences in this study were obtained from genomic data using a custom script. In addition, COI gene sequences of other species that closely related to *Colletes gigas* were downloaded from NCBI (see Zhou et al., 2020, Supplementary Material) (Supplementary Table 1), removing all duplicate COI sequences. These COI sequences were aligned with Muscle implemented within MEGA 6.05. The Phylogenetic analysis was inferred using IQ-TREE 1.6.12 (Nguyen, et al. 2015). Branch support was conducted with 1000 replicates of ultrafast likelihood bootstrap.

**Literature Cited**

Zhou, Q. -S., Luo, A., Zhang, F., Niu, Z. -., Wu, Q. -T., Xiong, M., Orr, M. C., & Zhu, C. -D. (2020). The first draft genome of the plasterer bee *Colletes gigas* (Hymenoptera: Colletidae: Colletes). *Genome Biology and Evolution*, *12*(6), 860–866. https://doi.org/10.1093/gbe/evaa090

Nguyen, L. -T, Schmidt, H. A., von Haeseler, A., & Minh, B. Q. (2015). IQ-TREE: a fast and effective stochastic algorithm for estimating maximum-likelihood phylogenies. *Molecular Biology and Evolution*, *32*(1), 268–274. http://doi.org/10.1093/molbev/msu300

**Supplementary Materials**


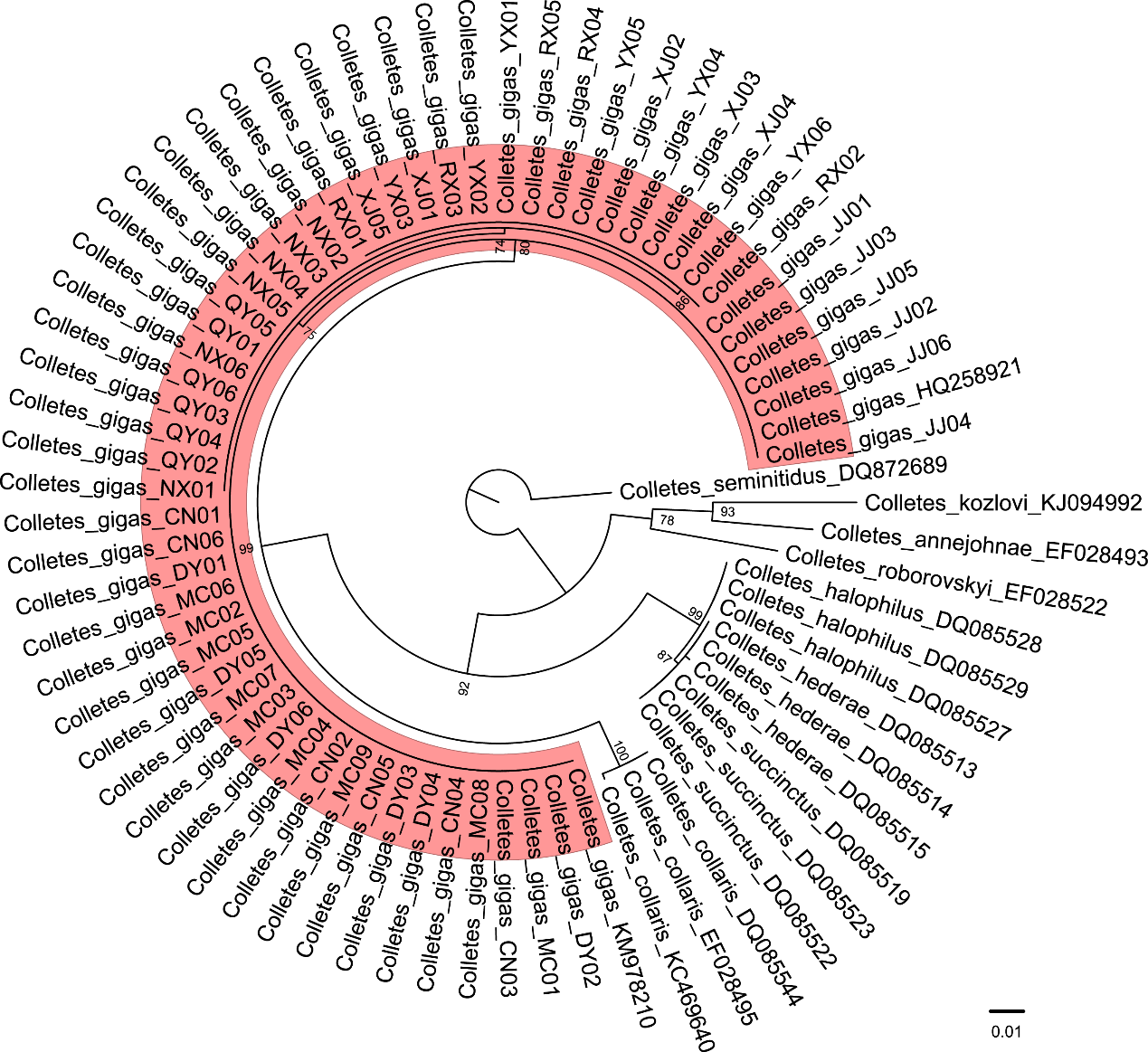


**FIGURE S1** Maximum likelihood phylogeny inferred from mitochondrial COI gene sequences available for *Colletes gigas* and some other closely related *Colletes* species from NCBI. The COI sequences of *C. gigas* are highlighted in light red.

**Supplementary Table 1** Summary of COI sequences used in this study

| Speceis | Genbank accession no. | Speceis | Genbank accession no. |
| --- | --- | --- | --- |
| *Colletes collaris* | DQ085544 | *Colletes succinctus* | DQ085519 |
| *Colletes collaris* | EF028495 | *Colletes succinctus* | DQ085522 |
| *Colletes collaris* | KC469640 | *Colletes succinctus* | DQ085523 |
| *Colletes halophilus* | DQ085527 | *Colletes kozlovi* | KJ094992 |
| *Colletes halophilus* | DQ085528 | *Colletes annejohnae* | EF028493 |
| *Colletes halophilus* | DQ085529 | *Colletes roborovskyi* | EF028522 |
| *Colletes hederae* | DQ085513 | *Colletes seminitidus* | DQ872689 |
| *Colletes hederae* | DQ085514 | *Colletes gigas* | KM978210 |
| *Colletes hederae* | DQ085515 | *Colletes gigas* | HQ258921 |

**S****upplementary Table 2** Sequencing and alignment

| Sample ID | Location | Collecting date | Latitude | Longitude | Altitude(m) | Depth |
| --- | --- | --- | --- | --- | --- | --- |
| DY01 | China: Guangdong, Dongyuan | 2019/11/30 | 24.1905 | 114.9792 | 168 | 36.30 |
| DY02 | China: Guangdong, Dongyuan | 2019/11/30 | 24.1905 | 114.9792 | 168 | 35.76 |
| DY03 | China: Guangdong, Dongyuan | 2019/11/30 | 24.1905 | 114.9792 | 168 | 35.58 |
| DY04 | China: Guangdong, Dongyuan | 2019/11/30 | 24.1905 | 114.9792 | 168 | 40.06 |
| DY05 | China: Guangdong, Dongyuan | 2019/11/30 | 24.1905 | 114.9792 | 168 | 37.89 |
| DY06 | China: Guangdong, Dongyuan | 2019/11/30 | 24.1905 | 114.9792 | 168 | 40.47 |
| YX01 | China: Fujian, Youxi | 2019/12/1 | 26.1719 | 118.2649 | 176 | 37.36 |
| YX02 | China: Fujian, Youxi | 2019/12/1 | 26.1719 | 118.2649 | 176 | 36.35 |
| YX03 | China: Fujian, Youxi | 2019/12/1 | 26.1719 | 118.2649 | 176 | 36.73 |
| YX04 | China: Fujian, Youxi | 2019/12/1 | 26.1719 | 118.2649 | 176 | 42.75 |
| YX05 | China: Fujian, Youxi | 2019/12/1 | 26.1719 | 118.2649 | 176 | 38.13 |
| YX06 | China: Fujian, Youxi | 2019/12/1 | 26.1719 | 118.2649 | 176 | 37.71 |
| CN01 | China: Zhejiang, Cangnan | 2019/11/2 | 27.4591 | 120.2556 | 401 | 34.88 |
| CN02 | China: Zhejiang, Cangnan | 2019/11/2 | 27.4591 | 120.2556 | 401 | 35.55 |
| CN03 | China: Zhejiang, Cangnan | 2019/11/2 | 27.4591 | 120.2556 | 401 | 35.48 |
| CN04 | China: Zhejiang, Cangnan | 2019/11/2 | 27.4591 | 120.2556 | 401 | 41.45 |
| CN05 | China: Zhejiang, Cangnan | 2019/11/2 | 27.4591 | 120.2556 | 401 | 39.77 |
| CN06 | China: Zhejiang, Cangnan | 2019/11/2 | 27.4591 | 120.2556 | 401 | 45.60 |
| XJ01 | China: Jiangxi, Xiajiang | 2019/12/3 | 27.6546 | 115.1285 | 73 | 36.68 |
| XJ02 | China: Jiangxi, Xiajiang | 2019/12/3 | 27.6546 | 115.1285 | 73 | 36.02 |
| XJ03 | China: Jiangxi, Xiajiang | 2019/12/3 | 27.6546 | 115.1285 | 73 | 38.23 |
| XJ04 | China: Jiangxi, Xiajiang | 2019/12/3 | 27.6546 | 115.1285 | 73 | 37.52 |
| XJ05 | China: Jiangxi, Xiajiang | 2019/12/3 | 27.6546 | 115.1285 | 73 | 37.61 |
| JJ01 | China: Jiangxi, Jiujiang | 2019/11/21 | 29.5333 | 116.0748 | 82 | 39.88 |
| JJ02 | China: Jiangxi, Jiujiang | 2019/11/21 | 29.5333 | 116.0748 | 82 | 37.74 |
| JJ03 | China: Jiangxi, Jiujiang | 2019/11/21 | 29.5333 | 116.0748 | 82 | 37.21 |
| JJ04 | China: Jiangxi, Jiujiang | 2019/11/21 | 29.5333 | 116.0748 | 82 | 40.40 |
| JJ05 | China: Jiangxi, Jiujiang | 2019/11/21 | 29.5333 | 116.0748 | 82 | 39.20 |
| JJ06 | China: Jiangxi, Jiujiang | 2019/11/21 | 29.5333 | 116.0748 | 82 | 39.71 |
| QY01 | China: Anhui, Qinyang | 2020/10/29 | 30.5977 | 117.8796 | 35 | 37.60 |
| QY02 | China: Anhui, Qinyang | 2020/10/29 | 30.5977 | 117.8796 | 35 | 36.80 |
| QY03 | China: Anhui, Qinyang | 2020/10/29 | 30.5977 | 117.8796 | 35 | 39.49 |
| QY04 | China: Anhui, Qinyang | 2020/10/29 | 30.5977 | 117.8796 | 35 | 40.94 |
| QY05 | China: Anhui, Qinyang | 2020/10/29 | 30.5977 | 117.8796 | 35 | 41.10 |
| QY06 | China: Anhui, Qinyang | 2020/10/29 | 30.5977 | 117.8796 | 35 | 39.54 |
| MC01 | China: Hubei, Macheng | 2019/11/10 | 31.5303 | 115.1678 | 218 | 52.59 |
| MC02 | China: Hubei, Macheng | 2019/11/10 | 31.5303 | 115.1678 | 218 | 39.22 |
| MC03 | China: Hubei, Macheng | 2019/11/10 | 31.5303 | 115.1678 | 218 | 45.13 |
| MC04 | China: Hubei, Macheng | 2019/11/10 | 31.5303 | 115.1678 | 218 | 41.24 |
| MC05 | China: Hubei, Macheng | 2019/11/10 | 31.5303 | 115.1678 | 218 | 37.43 |
| MC06 | China: Hubei, Macheng | 2019/11/10 | 31.5303 | 115.1678 | 218 | 41.18 |
| MC07 | China: Hubei, Macheng | 2019/11/10 | 31.5303 | 115.1678 | 218 | 36.07 |
| MC08 | China: Hubei, Macheng | 2019/11/10 | 31.5303 | 115.1678 | 218 | 43.61 |
| MC09 | China: Hubei, Macheng | 2019/11/10 | 31.5303 | 115.1678 | 218 | 36.12 |
| NX01 | China: Hunan, Ningxiang | 2019/11/19 | 27.9832 | 112.4206 | 117 | 38.64 |
| NX02 | China: Hunan, Ningxiang | 2019/11/19 | 27.9832 | 112.4206 | 117 | 36.80 |
| NX03 | China: Hunan, Ningxiang | 2019/11/19 | 27.9832 | 112.4206 | 117 | 38.84 |
| NX04 | China: Hunan, Ningxiang | 2019/11/19 | 27.9832 | 112.4206 | 117 | 37.04 |
| NX05 | China: Hunan, Ningxiang | 2019/11/19 | 27.9832 | 112.4206 | 117 | 39.82 |
| NX06 | China: Hunan, Ningxiang | 2019/11/19 | 27.9832 | 112.4206 | 117 | 40.16 |
| RX01 | China: Sichuan, Rongxian | 2019/11/14 | 29.4377 | 104.2913 | 542 | 37.05 |
| RX02 | China: Sichuan, Rongxian | 2019/11/14 | 29.4377 | 104.2913 | 542 | 37.26 |
| RX03 | China: Sichuan, Rongxian | 2019/11/14 | 29.4377 | 104.2913 | 542 | 37.30 |
| RX04 | China: Sichuan, Rongxian | 2019/11/14 | 29.4377 | 104.2913 | 542 | 36.13 |
| RX05 | China: Sichuan, Rongxian | 2019/11/14 | 29.4377 | 104.2913 | 542 | 39.15 |

**Supplementary Table 3** Cross-validation error result for varying values of *K* in the ADMIXTURE analysis

| Number of cluster | CV error value |
| --- | --- |
| CV error (*K*=2) | 0.49012 |
| CV error (*K*=3) | 0.49559 |
| CV error (*K*=4) | 0.5399 |
| CV error (*K*=5) | 0.56801 |
| CV error (*K*=6) | 0.61259 |
| CV error (*K*=7) | 0.65109 |
| CV error (*K*=8) | 0.70105 |
| CV error (*K*=9) | 0.76948 |
